# The *Myo*‐inositol pathway does not contribute to ascorbic acid synthesis

**DOI:** 10.1111/plb.12898

**Published:** 2018-09-24

**Authors:** E. Ivanov Kavkova, C. Blöchl, R. Tenhaken

**Affiliations:** ^1^ Department of Biosciences University of Salzburg Salzburg Austria

**Keywords:** Alternative *myo*‐inostiol pathway, ascorbic acid, glucuronic acid, mammalian‐like pathway, *myo*‐inositol

## Abstract

- Ascorbic acid (AsA) biosynthesis in plants predominantly occurs *via* a pathway with d‐mannose and l‐galactose as intermediates. One alternative pathway for AsA synthesis, which is similar to the biosynthesis route in mammals, is controversially discussed for plants. Here, *myo*‐inositol is cleaved to glucuronic acid and then converted *via* l‐gulonate to AsA. In contrast to animals, plants have an effective recycling pathway for glucuronic acid, being a competitor for the metabolic rate. Recycling involves a phosphorylation at C1 by the enzyme glucuronokinase.
- Two previously described T‐DNA insertion lines in the gene coding for glucuronokinase1 show wild type‐like expression levels of the mRNA in our experiments and do not accumulate glucuronic acid in labelling experiments disproving that these lines are true knockouts. As suitable T‐DNA insertion lines were not available, we generated frameshift mutations in the major expressed isoform glucuronokinase1 (At3g01640) to potentially redirect metabolites to AsA.
- However, radiotracer experiments with ^3^H‐*myo*‐inositol revealed that the mutants in glucuronokinase1 accumulate only glucuronic acid and incorporate less metabolite into cell wall polymers. AsA was not labelled, suggesting that *Arabidopsis* cannot efficiently use glucuronic acid for AsA biosynthesis.
- All four mutants in glucuronokinase as well as the wild type have the same level of AsA in leaves.

## Introduction

**Figure 1:**
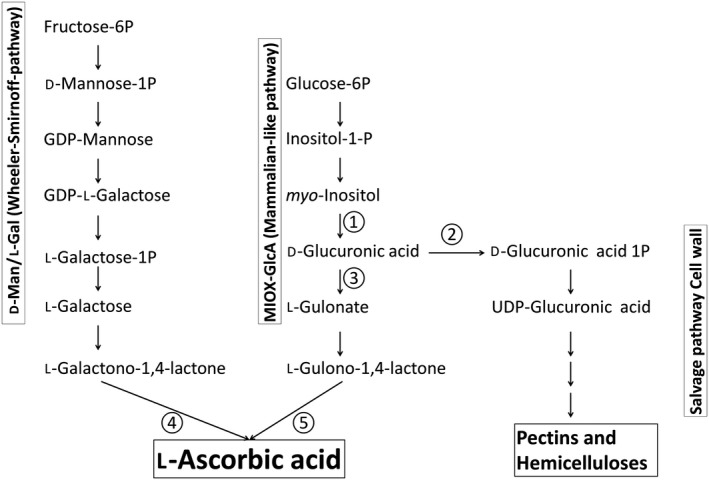
Pathway overview for AsA biosynthesis in plants. The d‐Man/l‐Gal pathway (left) is the dominant route to AsA in plants. The mammalian‐like pathway (middle) has been debated for many years and is addressed in this paper. The salvage pathway (right) contributes to cell wall polymers in many plants. ①: *myo*‐inositol oxygenase (MIOX); ②: glucuronokinase; ③: glucuronate reductase; ④: l‐galactono‐1,4‐lactone dehydrogenase (GLDH); ⑤: gulono‐1,4‐lactone oxidase.

Ascorbic acid (AsA) is an important antioxidant in plants and mainly involved in the removal of reactive oxygen species (ROS). Humans need to take up AsA with their food (vitamin C), which results in broad scientific interest in AsA biosynthesis and regulation in plants. The role of AsA for plants, the biosynthesis and attempts to increase AsA levels through transgenic approaches is covered by numerous comprehensive reviews and publications (*e.g*. Wheeler *et al*. 1998; Ishikawa *et al*. 2006). Early feeding experiments with radioactive precursors revealed that the biosynthesis of AsA follows different routes in plants and animals. The mammalian route involves UDP‐GlcA (UDP‐d‐glucuronic acid) to attach a GlcA sugar to an unknown metabolite, from which, through the action of a glucuronidase, GlcA is cleaved off (Linster & Van Schaftingen 2007). GlcA is then reduced to l‐gulonate, dehydrated to gulono‐1,4‐lactone and finally oxidised to AsA (Fig. 1). This route could not be confirmed for plants using tracer experiments (Loewus *et al*. 1956, 1962). Instead, it was found, that the label from *myo*‐inositol goes into hemicelluloses and pectins of the plant cell wall *via* GlcA and UDP‐GlcA (Loewus *et al*. 1962). New radiotracer feeding experiments revealed the AsA biosynthesis route in plants, in which d‐mannose is activated to GDP‐mannose, epimerised to GDP‐l‐galactose and hydrolysed to l‐galactose (Fig. 1). This metabolite undergoes lactone formation and is finally oxidised to l‐AsA (Wheeler *et al*. 1998). This route is backed up by mutants like *vtc1*,* vtc2* and *vtc5* which have reduced levels of AsA or the mutations are even lethal during embryogenesis (Conklin *et al*. 1997; John *et al*. 2007). Therefore the predominant route to AsA is the d‐Man/l‐Gal pathway, and the presence of alternative pathways has long been debated in plants (Smirnoff *et al*. 2001; Wheeler *et al*. 2015). The two favourite candidates are the d‐galacturonate pathway and a mammalian‐like pathway which use d‐glucuronate. The mammalian‐like pathway was suggested in two publications. In the first one, the enzyme *myo*‐inositol oxygenase (MIOX) was overexpressed in *Arabidopsis* resulting in a two‐ to three‐fold increase of AsA (Lorence *et al*. 2004). MIOX catalyses the oxygen‐dependent ring cleavage of *myo*‐inositol to GlcA. A follow‐up paper identified alkaline phosphatase acting on phytic acid as a possibility to increase AsA. The complete dephosphorylation of phytate releases *myo*‐inositol. When the phosphatase was overexpressed in *Arabidopsis* a twofold increase in AsA was observed (Zhang *et al*. 2008). The steady level of phytate is reduced by ca. 7.5 nmol·g·FW^−1^ in the overexpressor lines. The observed increase in AsA is ca. 3 μmol·g·FW^−1^. This would indicate that the flux through the phytate pathway must be 400 times higher than the flux of AsA turnover.

In independent experiments with the MIOX‐overexpressor lines, the increase of AsA could not be confirmed, although the flux from *myo*‐inositol into cell wall polymers was indeed increased (Endres & Tenhaken 2009). Furthermore, a quadruple knockout mutant in the whole MIOX gene family resulted in plants with the same AsA levels as measured in WT plants (Endres & Tenhaken 2011). The overexpression of MIOX genes in tomato (Cronje *et al*. 2012) or rice (Duan *et al*. 2012) also did not lead to increased AsA levels.

The mammalian‐like pathway to AsA starting from d‐GlcA requires three enzymes to catalyse the conversion of d‐GlcA into AsA. First, d‐GlcA is reduced to l‐gulonate by glucuronoreductase. The second step involves the formation of a lactone catalysed by aldonolactonase. The product l‐gulono‐1,4‐lactone is then finally oxidised to l‐AsA. Using bioinformatics Ruggieri *et al*. (2016) found some candidate genes in tomato for the reductase (first step ③ in Fig. 1) but no candidate gene for the second step. Two candidate genes were predicted for the final oxidase step (⑤ in Fig. 1). These data do not confirm enzyme activity or physiological relevance. Aboobucker *et al*. (2017) recently characterised two genes from *Arabidopsis* with l‐gulono‐1,4‐lactone oxidase activity. The enzymes have a high K_m_ (33 mm) and a very low k_cat_ (0.005·s^−1^). The terminal enzyme of the well‐established Wheeler‐Smirnoff pathway, l‐galactono‐1,4‐lactone dehydrogenase ④, is far more active, with a K_m_ of 0.17 mm and a k_cat_ of 134·s^−1^. This corresponds to a more than 25,000‐fold higher catalytic activity of ④ compared to ⑤. Maruta *et al*. (2010) overexpressed candidate genes for l‐gulono‐1,4‐lactone in tobacco BY2 cell cultures. They found no increase in AsA in overexpressing lines. Only after feeding high concentrations of l‐gulono‐1,4‐lactone (10 mm) did the BY2 cells have elevated levels of AsA. Aboobucker *et al*. (2017) also overexpressed the l‐gulono‐1,4‐lactonase genes in *Arabidopsis* without any increase in AsA levels.

Unfortunately, most of the papers describe the overexpression of genes as the starting point and the outcome in AsA levels at the end, without further measurements of metabolites which might support the data. Here we show that a frameshift mutant in the gene for glucuronokinase1 (*GlcAK1*) has a reduced flux of GlcA into cell wall polymers. Radiotracer experiments with *myo*‐inositol show no flux of labelled GlcA into the AsA pool but instead accumulate GlcA. Thus, GlcA cannot be metabolised to AsA. The level of AsA in these mutants is the same as in WT plants.

## Material and methods

### Plant material and growth conditions

In this work *Arabidopsis thaliana* (ecotype Columbia 0) was used as a WT plant. Previously described T‐DNA insertion mutants *glcak1‐1* (SALK_076931) and *glcak1‐2* (SALK_127949c) were obtained from the Arabidopsis Biological Resource Center and another two mutant lines *glcak1‐3* (contains CAS9 nuclease) and *glcak1‐4* were created by CRISPR/Cas9 technology. Plants were grown in a growth chamber in pots on standard soil (type ED73) under long‐day conditions with 16‐h light at 150 μmol·m^−2^·s^−1^. Temperature during the light phase was 23 °C and 18 °C in the dark. For RT‐PCR and labelling experiments seedlings were grown in liquid medium. Seeds were surface sterilised in ethanol and grown in 0.5 × MS (Basal Salt Mixture, Duchefa #M0245; Duchefa, Haarlem, the Netherlands), pH 5.7 (KOH), with 1 g·l^−1^ sucrose for 10 days. Then the medium was changed to 0.5 × MS (or 0.5 × MS with 0.37 MBq *myo*‐[2‐^3^H]‐inositol; final concentration in medium is 0.625 μm) for the next 3 days. For sugar measurements sterile seeds were incubated on 0.5 × MS (without micronutrients; Duchefa #M0221), pH 5.7 (KOH), plates with 0.8% plant agar. Harvested samples were immediately used for experiments or frozen in liquid nitrogen and stored at −80 °C until used.

### Real‐time PCR

Total RNA was isolated from 13‐day‐old seedlings using Tri‐Reagent method. The aqueous phase containing the RNA was further purified on a silica spin column and eluted in 40 μl DEPC‐H_2_O. Subsequently, RNA was converted to cDNA by RevertAid Reverse Transcriptase (Thermo Scientific, Waltham, MA, USA) using an anchored oligo(dT) primer. Real‐time PCR was performed with a Mx3000P qPCR system (Stratagene, San Diego, CA, USA) and PCR products were detected by SYBR green fluorescence. The obtained values were analysed using the 2^−ΔΔCT^ method (Livak & Schmittgen 2001). RT‐PCR primers are listed in Table S1.

### Resequencing of the position of the T‐DNA insertion in *GlcAK1* mutants

Position of the T‐DNA insertion in *glcak1‐1* and *glcak1‐2* was determined by direct sequencing of PCR products obtained with primers binding to the left border of the T‐DNA insertion and on exon1 of *GlcAK1* (Table S1). Genomic DNA was isolated from a leaf of a 4‐week‐old plant by standard methods and 1 μl was used as a DNA template for PCR (reaction volume 30 μl). PCR product was purified with the GeneJET PCR Purification Kit (Thermo Scientific).

### Construction of CRISPR/Cas9 knockouts

Mutants in *GlcAK1* were generated using CRISPR/Cas9 technology (Fauser *et al*. 2014). *A. thaliana* (ecotype Columbia 0) were transformed with the CRISPR/Cas9 construct by *Agrobacterium tumefaciens* (strain GV3101) according to direct‐dip protocol (Davis *et al*. 2009). Plants with a homozygous mutation in *GlcAK1* were selected in T_2_ generation by sequencing of a PCR product spanning the targeted region of exon1 (Table S1). In this study, two lines, *glcak1‐3* and *glcak1‐4*, were used.

### Feeding of seedlings with ^3^H‐*myo*‐inositol followed by metabolite separation on HPLC

Ten‐day‐old seedlings labelled with *myo*‐[2‐ ^3^H]–inositol for 3 days were washed twice with 0.5 × MS medium containing 8 mm inositol to exchange non‐specifically bound ^3^H‐*myo*‐inositol. The seedlings were carefully dried with a soft cosmetic tissue and snap frozen in N_2_ with two 3 mm stainless steel balls. Seedlings were homogenised in a liquid N_2_ cooled ball mill to a fine powder. The homogenate was incubated in 250 μl methanol:chloroform (7:3) for 2 h at 4 °C and vortexed several times in between. A total of 350 μl H_2_O was added and samples were incubated in a cooled shaker for 10 min. After centrifugation, the upper phase was transferred to a new reaction tube and dried in a vacuum centrifuge. The dry pellet was re‐dissolved in 40 μl H_2_O. 10 μl of this sample were diluted with 100 mm NH_4_‐acetate and applied to a Hilic column (125 × 4 mm Nucleodur 100‐5; Machery‐Nagel, Düren, Germany). HPLC analysis was performed with 100 mm NH_4_‐acetate (buffer A) and acetonitrile (buffer B) using isocratic conditions (80% B; flow rate 0.6 ml·min^−1^). Samples were collected using a fraction collector, mixed with 2 ml scintillation cocktail (Rotiszint eco plus; Carl Roth, Karlsruhe, Germany) and counted. Standard compounds (AsA, *myo*‐inositol and GlcA) were separated under the same conditions. Ascorbic acid was detected using UV light (262 nm) whereas *myo*‐inositol was determined with an enzyme assay (Megazyme #K‐INOSL; Megazyme, Wicklow, Ireland). GlcA was determined with the hydroxy‐benzoic acid hydrazide assays for aldehyde groups (5% HBH dissolved in 0.5 m HCl, diluted 1:10 in 0.5 m NaOH directly before use). Fractions from the HPLC separation were dried in a vacuum centrifuge, dissolved in 200 μl HBH reagent and boiled for 10 min. The OD_410 nm_ was read in a plate reader.

Incorporation of ^3^H sugars into cell walls was determined after extraction of ground seedling material, twice with 70% ethanol followed by a chloroform:methanol (1:1) extraction and an acetone extraction step. The pellet corresponding to crude cell walls was air‐dried and counted with 2 ml scintillation cocktail (Rotizint eco plus; Carl Roth, Karlsruhe, Germany). In some cases, the lower phase of the metabolite sample, containing the insoluble fraction of the cells, was further extracted with 70% ethanol as described above.

### Measurements of GlcA levels in seedlings

Free GlcA was extracted from seedlings using methanol:chloroform (7:3) extraction as described above. The dried metabolites were dissolved in 200 μl H_2_O and separated on a CarboPac PA20 column (150 × 3 mm) on an ICS3000‐HPLC system: Buffer A: 200 mm NaOH; buffer B: 15 mm NaOH; buffer C: 50 mm NaOH and 500 mm Na‐acetate; Flow rate 0.45 ml·min^−1^; t0: 100%B; t12: 100% B; t12.1: 60% B and 40% C; t20 60% B and 40% C.

### Ascorbic acid measurements

The AsA was measured from individual leaves of 4‐week‐old plants. The fresh weight was determined, and the leaf was then immediately homogenised in 1 ml 1 m HClO_4_ and sea sand in a small cooled mortar. The homogenate was transferred to a reaction tube, centrifuged for 2 min, and 500 μl of the clear supernatant was neutralised to pH 5 by addition of 42 μl 5 m K_2_CO_3_ and 200 μl HEPES‐KOH buffer 0.1 m pH 7. The sample was briefly stored on ice and precipitated KClO_4_ removed by centrifugation for 2 min. 100 μl of this sample were mixed with 900 μl Na‐P_i_ buffer pH 5.6 and the OD_262 nm_ determined. One unit of ascorbate oxidase (Applichem, Darmstadt, Germany) was added to the assay and carefully mixed. After 3 min, the OD_262 nm_ reached a stable value. The difference between the two values corresponds to reduced AsA. For total AsA, 200 μl of the neutralised extract were mixed with 200 μl NaP_i_ buffer (100 mm pH 7.5) and 20 μl TCEP (25 mm) and incubated at room temperature for 30 min. The amount of total AsA was measured with 200 μl extract as described above.

## Results

The conflicting data on the role of a mammalian‐like pathway in AsA synthesis in plants was investigated in knockout mutants of glucuronokinase1 (GlcAK1). It has previously been shown that GlcAK is part of a salvage pathway for GlcA leading *via* GlcA‐1P to UDP‐GlcA, a precursor for roughly half of the biomass of primary cell walls in *Arabidopsis* (Pieslinger *et al*. 2010). We therefore hypothesised that blocking the pathway to GlcA‐1P should potentially redirect GlcA to AsA *via* a mammalian‐like pathway (Fig. 1).

### Verification of T‐DNA insertional mutants *glcak1‐1* and *glcak1‐2*

**Figure 2:**
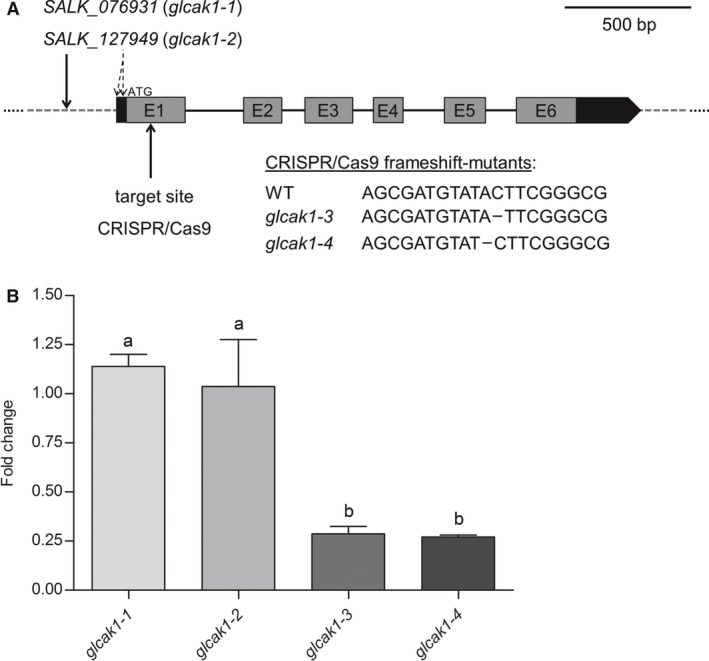
Gene structure, position of T‐DNA insertion in GlcAK1 and expression of the *GlcAK1*‐gene. A: T‐DNA position for both SALK lines was determined by sequencing and found to be 197 bp upstream of the start ATG. The T‐DNA position according to Xiao *et al*. (2017) is shown with dashed lines. The position and the sequences of the frame shift mutants *glcak1‐3* and *glcak1‐4* in exon1, generated by CRISPR/Cas9, is also shown. UTRs are indicated in black and exons in grey boxes, introns are represented by black lines. B: Expression of *GlcAK1* was measured by qPCR for the four *glcak1* mutants. The data show average expression from three biological independent experiments. Statistical differences were evaluated using anova (Tukey's test, *P* < 0.01), different letters display significant differences between lines.

To study an influence of *GlcAK1* knockout on the mammalian‐like pathway, previously described T‐DNA insertional mutants *glcak1‐1* and *glcak1‐2* (Xiao *et al*. 2017) were chosen (Fig. 2A). However, when relative *GlcAK1* expression in mutant lines was compared to WT, no differences were found (Fig. 2B). To confirm the position of the T‐DNA insertion, both lines were verified by sequencing. The T‐DNA insertions was found in the promoter region, which differed in the two mutants from the positions previously described (Xiao *et al*. 2017), but corresponded to the locations found in the databases (http://signal.salk.edu/cgi-bin/tdnaexpress; Arabidopsis Information Resource (TAIR)) (Fig. 2A). The transcription level of *GlcAK1* was not decreased, probably due to the position of the T‐DNA insertion, which was detected in the promoter region 197 bp upstream of the ATG start codon (Fig. 2B).

### Preparation of new *GlcAK1* mutants with CRISPR technology

In order to obtain plants with a loss of GlcAK1 activity, CRISPR/Cas9 mutants were prepared. The gRNA was targeting a sequence in exon1. In the T_2_ generation, several plants with a homozygous mutation were identified. For this study, *glcak1‐3* and *glcak1‐4* containing a frameshift mutation resulting in a premature stop codon close by were used. Both lines showed a decreased transcript level of *GlcAK1*, around 28% of the WT values (Fig. 2B), possibly because the non‐translated part of the mRNA makes it more sensitive to degradation.

### Seedlings of CRISPR/Cas9 mutants *glcak1‐3* and *glcak1‐4* accumulate GlcA after ^3^H‐*myo*‐inositol feeding

**Figure 3:**
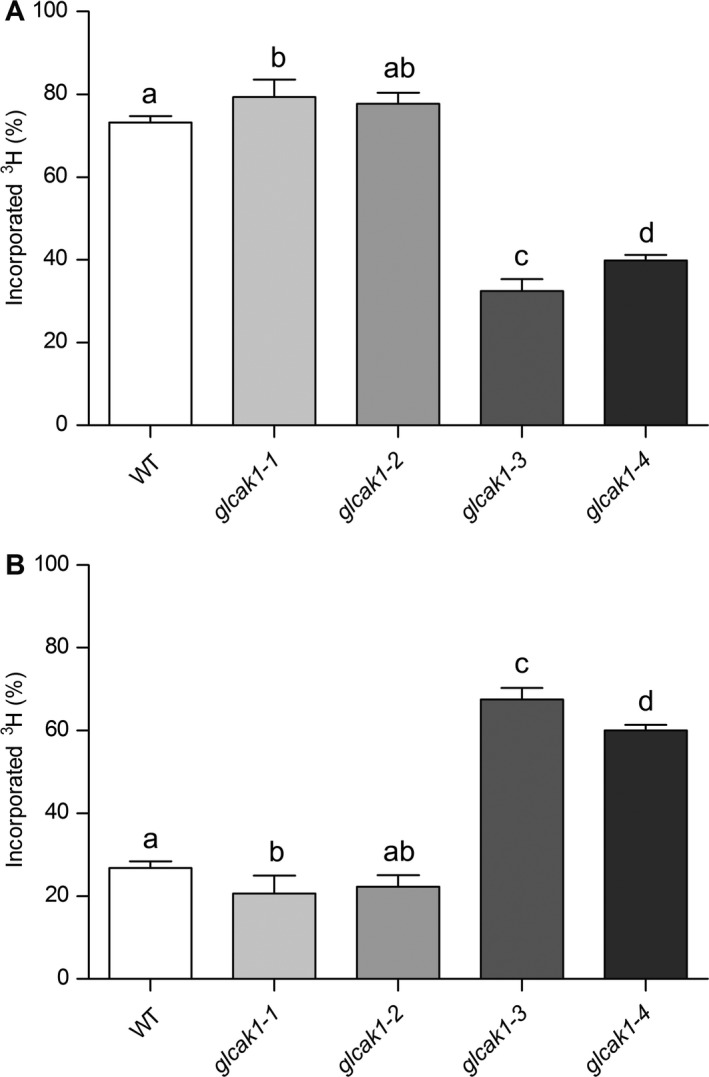
Distribution of radiolabelled compounds after ^3^H‐*myo*‐inositol feeding. Seedlings were grown in 0.5 × MS medium and ^3^H‐*myo*‐inositol was added for 3 days. A: Radioactivity from insoluble cell wall, and B: ethanol soluble metabolites determined by scintillation counting. Statistically significant differences were determined using anova (Tukey's test, *P* < 0.01), different letters display significant differences (*n* = 6).

To investigate whether the mutation in GlcAK1 redirects GlcA from the MIOX pathway to AsA, ^3^H‐labelled *myo*‐inositol was fed to 10‐day‐old *Arabidopsis* seedlings. After 3 days of feeding, radioactivity in soluble and cell wall fractions was measured. As expected, in WT and the SALK mutants (*glcak1‐1*;* glcak1‐2*) the main portion of the label was incorporated into cell walls (73–79%; Fig. 3A). In contrast, the CRISPR/Cas9 frameshift‐mutants (*glcak1‐3*;* glcak1‐4*) showed reduced values of label in cell walls (32–40% incorporated ^3^H), whereas labelled soluble metabolites are much more abundant in *glcak1‐3* and *glcak1‐4*. The SALK mutants and WT had only 21–27% of the label, but *glcak‐3* and *glcak‐4* retained 68% and 60% label in the soluble fraction, respectively (Fig. 3B). As we have generated knockout mutants only in the major expressed isoform *GlcAK1*, certain amounts of label go into the cell wall fraction *via* the second isoform of glucuronokinase.

**Figure 4:**
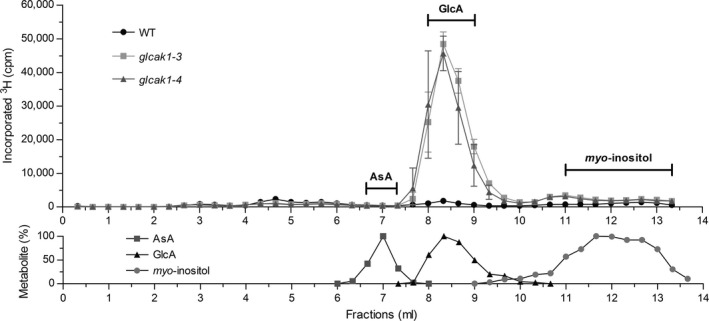
Profile of ^3^H‐labelled compounds after separation on a Hilic HPLC column. Soluble metabolites after ^3^H‐*myo*‐inositol feeding (as described in Fig. 3) were subjected to HPLC separation. Fractions of 0.33 ml were collected in a microtiter plate and counted. The experiment was done with *glcak1‐3* and *glcak1‐4* mutants, showing the same metabolite pattern. The elution profile of AsA was determined by UV absorption. GlcA was measured by aldehyde colour assay and *myo*‐inositol was quantified by an enzymatic reaction.

In order to identify the ^3^H‐labelled metabolites derived from inositol feeding, soluble fractions of WT and *glcak1‐3* and *glcak1‐4* were further separated by HPLC on a HILIC column and eluates were collected in 0.33‐ml fractions (Fig. 4). The HILIC column was chosen as it allowed the separation of the three metabolites of interest, *myo*‐inositol, GlcA and AsA. All samples were measured by scintillation counting. Surprisingly, the largest portion of the label was found in the GlcA peak, where *glcak1‐3* and *glcak1‐4* mutants accumulated around 26 times more label than WT plants. We cannot fully exclude that some of label is also present in l‐gulonate, the product of the enzyme glucurono reductase if this metabolite would co‐elute with d‐glucuronic acid. l‐gulonate neither absorbs UV light nor has an aldehyde group, which we would need to detect this compound. Whether the enzyme glucoronate reductase exist in plants cannot easily be answered by bioinformatics. This enzyme belongs to a larger gene family of conserved aldehyde reductases, which, however, act on various but diverse substrates. The gene from mouse was recently identified and the enzymatic function was confirmed in knockout mice (Takahashi *et al*. 2012). In fractions containing AsA, no increased radioactivity was detected in any plant line (Fig. 4). The data show that *myo*‐inositol is almost quantitatively converted to GlcA, which strongly increases in concentration when the glucuronokinase is blocked but a redirection of GlcA into other pathways including the formation of AsA does not occur. Loewus (1963) used two different isotopes of *myo*‐inositol, labelled either at [2‐^3^H] or [2‐^14^C]. They did not find labelled AsA after feeding both forms of radioactive *myo*‐inositol, consistent with our results.

### The CRISPR/Cas9 mutants *glcak‐3* and *glcak‐4* accumulate GlcA

**Figure 5:**
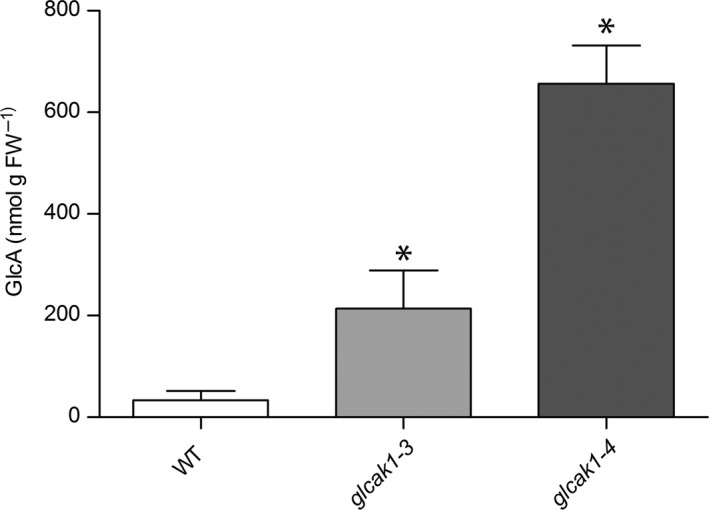
Metabolite analysis of WT,* glcak1‐3* and *glcak1‐4* mutants. Metabolites were separated on a CarboPac PA20 column to quantify GlcA. The data show average ± SD of three independent biological experiments. Mutants were compared to WT using *t*‐test (two‐tailed, unpaired, *P* < 0.01), significant differences are displayed with *.

To further test whether GlcA accumulates also under normal growth conditions, GlcA concentration in WT and *glcak1‐3* and *glcak1‐4* mutants was measured in seedlings grown on 0.5 × MS agar plates. Both mutants showed a 6–19 times higher concentration of GlcA compared to WT (Fig. 5). This finding suggests that glucuronoreductase has either very low activity or is absent in plants. The concentration of GlcA in metabolite extracts from WT plants is rather low.

### Measurement of AsA in leaves

**Figure 6:**
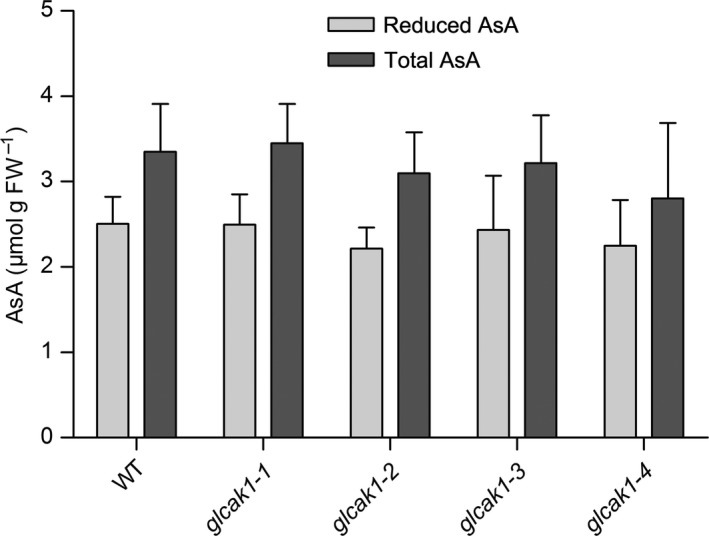
Measurement of AsA from WT and *glcak1* mutants. Leaves were taken from plants at different times of day and immediately measured for AsA. The data show average values ± SD from eight samples. There were no statistical differences between lines using anova (Tukey's test, *P* < 0.05).

Ascorbic acid levels in leaves of 4‐week‐old plants were analysed at different time points of the day. There was no significant difference between WT and all *glcak1* mutants (Fig. 6). The amount of reduced AsA is also highly similar between all genotypes.

## Discussion

Ascorbic acid is an important antioxidant in plants and animals for which a few different biosynthetic pathways have evolved. Here we address the long‐debated question whether a mammalian‐like pathway for AsA is functional in *Arabidopsis*, which was proposed in some previous publications (Lorence *et al*. 2004; Zhang *et al*. 2008). The concept of the mammalian‐like pathway needs free GlcA as precursor, which is then converted to l‐gulonate, l‐gulono‐1,4‐lactone and finally oxidised to AsA. A clear difference in the concept between plants and animals is the source of GlcA. Whereas mammals use GlcA, which is assumed to be derived from the hydrolysis of a glucuronylated compound in the liver (Linster & Van Schaftingen 2007), the suggested source of GlcA in plants is the oxygenative ring cleavage of *myo*‐inositol by the enzyme MIOX. Another important difference between plants and animals is a salvage pathway for GlcA in plants, which proceeds *via* GlcA‐1P to UDP‐GlcA. This pathway is absent in mammals (Pieslinger *et al*. 2010; Gangl *et al*. 2014). The salvage pathway for GlcA is well established and functional in plants, as indicated by several radioactive labelling experiments, in which the flux from *myo*‐inositol into cell wall material was found (Loewus *et al*. 1962; Seitz *et al*. 2000; Kanter *et al*. 2005; Endres & Tenhaken 2011). Furthermore, *ugd2,3* mutants in the biosynthesis of UDP‐GlcA, which have a defect in the formation of UDP‐GlcA *via* UDP‐Glc by the enzyme UDP‐glucose dehydrogenase, show a severe root phenotype (Reboul *et al*. 2011). The short roots and the defects during development can be largely rescued by feeding *myo*‐inositol or GlcA, which *via* the salvage pathway provides UDP‐GlcA to the plant mutants. So, if plants would also use GlcA for AsA biosynthesis, there would be competition between the cell wall pathway *via* glucuronokinase and the hypothetical conversion to l‐gulonate and further to AsA.

We have generated frameshift knockout mutants (*glcak1‐3*;* glcak1‐4*) in the *GlcAK1* gene as several of the available T‐DNA lines did not have changed relative gene expression compared to WT when tested. When low concentrations of ^3^H‐*myo*‐inositol was fed to WT and the *glcak1‐3* or *glcak1‐4* mutants a clear difference was found in the labelled products. Whereas less ^3^H label is present in the cell wall fraction of *glcak1‐3/1‐4*, a massive accumulation occurred in the soluble metabolites. A detailed HPLC analysis of the soluble products clearly shows that the label accumulates as GlcA in *Arabidopsis*. The inositol is quantitatively converted to GlcA by the MIOX enzymes but the use for cell wall biosynthesis thereafter is partially blocked in *glcak1‐3* and *glcak1‐4*. If plants use GlcA for AsA biosynthesis we would have expected to find the label in AsA rather than its accumulation in GlcA. The amount of fed ^3^H‐inositol is less than 1 μM excluding a perturbation of the metabolism by overloading the pathway. AsA is typically present in concentrations higher than 1000‐fold (low mM range). Here we show that the flux from *myo*‐inositol to AsA does not occur in *Arabidopsis*, because accumulated GlcA cannot be converted to AsA. The Loewus group came to a similar outcome, but their data could also be explained by the predominance of the salvage pathway to cell wall precursors (Loewus *et al*. 1962). The possible explanation for the accumulation of label in GlcA might be due to absence of glucuronate reductase, which is responsible for the conversion to l‐gulonate. To our knowledge, there are no clear data for the presence of this enzyme in plants.

The studies of Lorence *et al*. (2004) and Zhang *et al*. (2008) rely on a pathway which, according to the data presented in this paper, does not exist is *Arabidopsis*. Furthermore, AsA measurements in the same MIOX4‐overexpressing plants as Lorence *et al*. showed no clear differences in the AsA concentration between WT and the transgenic lines (Endres & Tenhaken 2009). The differences in the results for the same MIOX4‐overexpressing plants as in Lorence *et al*. remain difficult to explain. One aspect is the concentration of *myo*‐inositol in the transgenic lines, which is different between WT and the MIOX4‐overexpressors (Endres & Tenhaken 2009). Changes in the concentration of *myo*‐inositol are associated with changes in galactinol, a dimer of galactose and *myo*‐inositol. Galactinol was recently associated with stress gene expression in tobacco (Kim *et al*. 2008). The studies of Lorence *et al*. (2004) and Zhang *et al*. (2008) provide no direct link between higher transcript levels for MIOX4 and higher levels of AsA.

Inspired by the publication of Lorence *et al*. (2004), other research groups have also overexpressed MIOX genes, for instance in tomato (Cronje *et al*. 2012) or rice (Duan *et al*. 2012), to increase the AsA level in these crops. None of the transgenic plants contained higher levels of AsA than the WT controls, which is in good agreement with our data. If one includes the labelling studies from Loewus *et al*. (1962), it can be concluded that a mammalian‐like pathway to AsA is not functional in a diverse group of plants including a monocot.

Other groups have addressed the different pathways to AsA by searching for conserved biosynthesis genes (Wheeler *et al*. 2015; Ruggieri *et al*. 2016). These studies show that, for example, the enzymes gluconolactonase, as well as l‐gulonolactone oxidase, are absent in the genomes of higher plants. Moreover, there were no expressed sequence tags for gluconolactonase in kiwifruit (*Actinidia* spp.), which is one of the most suitable candidates to study mammalian‐like pathway as it is rich in AsA content and has much higher *myo*‐inositol concentrations than other plants (Bieleski *et al*. 1997; Crowhurst *et al*. 2008). Taken together, the information on the part of the mammalian‐like pathway starting from GlcA is either ambiguous or the evidence for particular genes is missing. There is therefore a need to confirm the data biochemically to prove the function of the enzymes and their biological relevance.

Xiao *et al*. (2017) recently published experiments about a knockout in *GlcAK1* and showed some changes in stress response, different expression of sugar‐related and ABA‐response genes. The changes were attributed to the knockout of the *GlcAK1* gene. We have tested the same T‐DNA lines (*glcak1‐1* and *glcak1‐2*) in our feeding experiments, but neither T‐DNA line show accumulation of GlcA as found in the frameshift mutations *glcak1‐3/1‐4* (compare Fig. 4). The paper of Xiao *et al*. (2017) suggests a T‐DNA insertion position close to the ATG start codon, which we cannot confirm. Resequencing of the DNA insertion position by us revealed a position 197 bp upstream of the ATG start codon, which is identical to the position shown on the T‐SIGNAL webpage (http://signal.salk.edu/cgi-bin/tdnaexpress). We also measured relative GlcAK1 expression with the *glcak1‐1* and *glcak1‐2* mutants, showing no difference between WT and mutant lines. This also explains why the flux of ^3^H‐*myo*‐inositol into cell walls is very similar in WT, *glcak1‐1* and *glcak1‐2* mutants.

Plants have established recycling pathways for many sugars, including GlcA. Mutations in the gene for GlcAK1 already lead to a significant increase in GlcA, although a second isoform exists in the *Arabidopsis* genome. The flux of ^3^H label from *myo*‐inositol into the cell wall is also detected in *glcak1‐3* and *glcak1‐4* mutants, although at a lower level, which confirms the function of GlcAK2 (At5g14470) as a second isoform of GlcAK. The paper of Zhao *et al*. (2013), however, claims a biological function of this gene as a galactokinase rather than a glucuronokinase. This is highly unlikely as the only galactokinase in *Arabidopsis* is encoded by a different gene (At3g06580; Egert *et al*. 2012). A possible misinterpretation of the gene ontology terms might have caused the wrong annotation and questionable conclusions of the paper. In fact, we have confirmed the function of GlcAK2 as a true glucuronokinase in preliminary experiments with purified recombinant enzyme from transient expression in tobacco plants.

## Conclusion

The mammalian‐like pathway to AsA *via myo*‐inositol and GlcA was proposed in previous publications. Here we show that a knockout in glucuronokinase1 reduces the flux of GlcA into cell wall polymers and leads to an accumulation of GlcA. We also found no evidence that GlcA is further used to synthesise AsA in *Arabidopsis*. Moreover, any direct evidence for a mammalian‐like pathway to AsA in plants is lacking.

## Supporting information


**Table S1.** Primers used in this study.
