# Supplementary material for: The Myo‐inositol pathway does not contribute to ascorbic acid synthesis
Source: Plant Biol (Stuttg). 2018 Sep 24;21(Suppl Suppl 1):95–102. doi: 10.1111/plb.12898 (PMC6492119; doi:10.1111/plb.12898)
Supplement: Supplementary file 1 — Table S1. Primers used in this study. [file PLB-21-95-s001.docx]

| **Primers** | **Forward (FW)**  **Reverse (RV)** |
| --- | --- |
| T-DNA insertion verification | FW: ACTCAACCCTATCTCGGGCTATTC  RV: CGAGATCATGGAATGGATGAG |
| Crispr/CAS9 gRNA | AACCCGAGCGATGTATACTT |
| Crispr/CAS9 mutation detection –PCR product | FW: GGGAGCTCAAAACGCGTCAGGGAAGAGAAG  RV: ACCACCATAGACCTGAGCAACC |
| Crispr/CAS9 mutation detection – sequencing | -  RV: CGAGATCATGGAATGGATGAG |
| RT-PCR *GlcAK1* | FW: GCAAGGAGCACATGGATAAATTGG  RV: GCCGAACCATACTATGTACCTTCC |

Table S1: Primers used in this study.
